# Flight behaviour monitoring and quantification of aedes aegypti using convolution neural network

**DOI:** 10.1371/journal.pone.0284819

**Published:** 2023-07-20

**Authors:** Nouman Javed, Prasad N. Paradkar, Asim Bhatti

**Affiliations:** 1 Institute for Intelligent Systems Research and Innovation, Deakin University, Geelong, Victoria, Australia; 2 CSIRO Health & Biosecurity, Australian Centre for Disease Preparedness, Geelong, Victoria, Australia; International Institute of Information Technology, INDIA

## Abstract

Mosquito-borne diseases cause a huge burden on public health worldwide. The viruses that cause these diseases impact the behavioural traits of mosquitoes, including locomotion and feeding. Understanding these traits can help in improving existing epidemiological models and developing effective mosquito traps. However, it is difficult to understand the flight behaviour of mosquitoes due to their small sizes, complicated poses, and seemingly random moving patterns. Currently, no open-source tool is available that can detect and track resting or flying mosquitoes. Our work presented in this paper provides a detection and trajectory estimation method using the Mask RCNN algorithm and spline interpolation, which can efficiently detect mosquitoes and track their trajectories with higher accuracy. The method does not require special equipment and works excellently even with low-resolution videos. Considering the mosquito size, the proposed method’s detection performance is validated using a tracker error and a custom metric that considers the mean distance between positions (estimated and ground truth), pooled standard deviation, and average accuracy. The results showed that the proposed method could successfully detect and track the flying (≈ 96% accuracy) as well as resting (100% accuracy) mosquitoes. The performance can be impacted in the case of occlusions and background clutters. Overall, this research serves as an efficient open-source tool to facilitate further examination of mosquito behavioural traits.

## Introduction

According to the World Health Organisation (WHO), mosquito-borne diseases are the most dangerous diseases among all vector-borne diseases [1], mainly due to the sheer number of people affected. Mosquito-borne diseases such as malaria, dengue, and yellow fever impact human health with high morbidity and mortality. These pathogens also affect the behaviours of mosquitoes [2], including locomotion [3–5], oviposition preferences [6], fertility [7] and feeding [8, 9]. Moreover, recent research has shown that vector-borne viruses can also infect and significantly impact the vector nervous system [10–12]. Monitoring mosquitoes’ flight trajectories can help in understanding and defining their locomotion behaviour, which can ultimately assist in determining their fitness, improving existing epidemiological models [13] and developing effective mosquito traps [14].

Initially, mosquitoes’ behaviours were based on manual observations by researchers [15, 16]. However, this is a very resource-expensive method and limits the number of individuals that can be simultaneously monitored. Moreover, some behavioural investigations require continuous observations, making the monitoring process laborious and time-consuming [17, 18]. Recently, the development of high-quality cameras has made automatic monitoring of objects possible through object techniques [19]. However, the object detection methods assume that the objects of concern in each frame are of significant size and have high contrast relative to the background [20]. In reality, the mosquitoes are smaller in size and have seemingly arbitrary moving patterns with speed variations [21]. In addition to these challenges, mosquitoes also depict different shapes by exhibiting different poses through random motion [22].

In recent times, artificial intelligence (AI) has played a vital role in transforming visualisation [23–26]. AI mimics human intelligence procedures through different algorithms built into a dynamic computing environment [27]. It consists of several subsets, including machine learning (ML), natural language processing (NLP), expert systems, and computer vision [28]. Machine learning focuses on building programs that learn from data and improve their accuracy automatically over time [29]. Machine learning can be divided into unsupervised and supervised learning. In supervised learning, machine programs learn the relations between inputs and outputs through the analysis of defined outputs of interest [30]. In contrast, unsupervised learning learns relations in data without depending on the external association of interest definitions [31].

Deep learning is the subset of machine learning and has set exciting new trends in machine learning over the years. In deep learning, machines are programmed to learn relations based on large quantities of raw data [32]. One important subset of AI is computer vision. Computer vision mimics human visual perception and reasoning capabilities [33]. Modern computer vision techniques heavily rely on machine learning and, specifically, deep learning algorithms. Over the decade, many algorithms and techniques have been developed to detect and monitor using computer vision. Region-based Convolutional Neural Networks (RCNN) models are among them and have played a key role in object detection.

In the past, machine learning-based models have been employed in finding different aspects of mosquitoes, such as detecting breeding grounds [34, 35] and identifying gender [36]. In addition, machine learning applications have also been reported in mosquito control [37]. There are also some commercially available tools for detecting mosquito flight behaviour [38]. However, to the best of the authors’ knowledge, no research has been found where machine learning models were used in tracking the trajectory of tiny flying objects like mosquitoes. These days, machine learning models are being used to detect different small objects, such as cell nuclei [39], showing the capability of machine learning models. Considering the potential of machine learning algorithms, it is hypothesised that machine learning based models can also help in tracking the trajectories of flying mosquitoes.

Taking into consideration the importance of understanding mosquitoes’ behavioural activities and the unavailability of any open-source mosquito detection and tracking tool, a method using the Mask RCNN algorithm and spline interpolation is presented here, which can efficiently detect mosquitoes and track their trajectories with higher accuracy. Additionally, it does not require any special high-quality setup and works excellently, even on low-resolution videos.

## Materials and methods

### Mosquitoes maintenance

*Aedes aegypti* mosquito colonies originating from Brisbane (provided by Prof. Ary Hoffman) were kept in the laboratory. Mosquitoes were maintained by artificial blood-feeding with chicken blood. Colony temperature was maintained at 27°C with humidity ranging between 60–70% under diurnal day: night (12h:12h) light cycle.

### Cage and feeding

**Fig 1:**
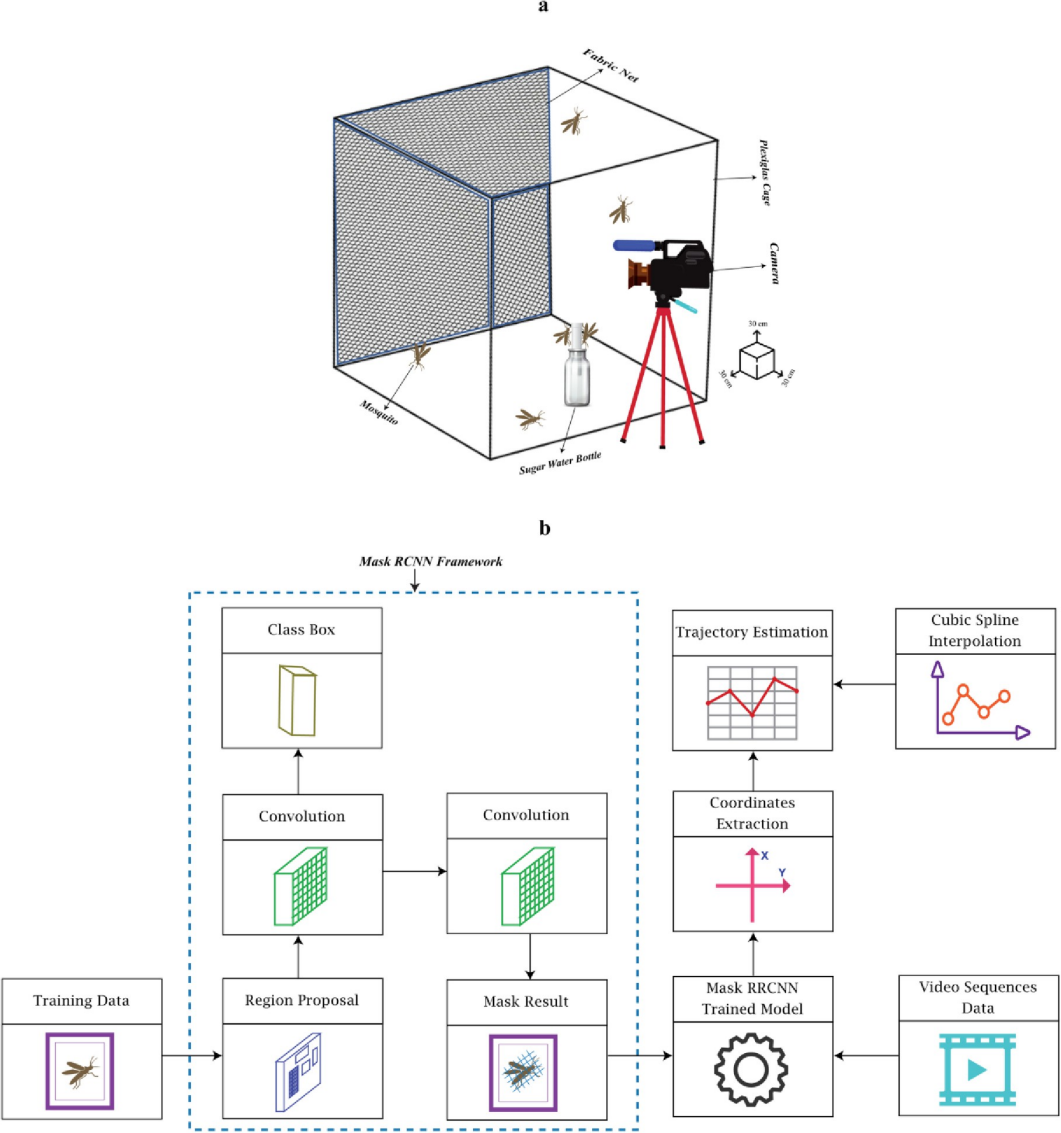
Experimental setup and trajectory estimation methodology. (a) The experimental setup consisted of a plexiglass cage, fabric net, sugar water bottle, mosquitoes, and camera. The number of mosquitoes in each recording was different. (b) The trajectory estimation was based on the mask RCNN framework and cubic spline interpolation. The training images data was fed into the Mask RCNN framework. Mask RCNN consists of RoIAlign to preserve spatial information. RoIAlign uses binary interpolation, which creates fix size feature map. RoIAlign layer output is fed into the mask head, which is consisted of two convolutional layers. Through this, masks are generated for each ROI, thus pixel to pixel segmentation of the images. Then video sequence data were processed using the trained model, and coordinates were extracted. Finally, the cubic spline interpolation was applied to fill the missing data smoothly.

*Aedes aegypti* females were kept in a transparent plexiglass cage of dimensions 30×30×30 cm for video recording. Mosquitoes were provided with sugar water *ad libitum* Fig 1A.

### Mosquitoes data recording and selection

The recording was started after one-week post-emergence, capturing six videos in total consisting of different mosquito batches and having a duration of around 1 minute each. Images from 2 videos with 5 and 24 mosquitoes were used to extract the training images, while images from 1 video having five mosquitoes were used to get the validation images. The remaining three videos with mosquitoes ranging from 5 to 27 were used for testing purposes. The videos were recorded under lights using the Flea3 camera [40]. From testing videos, three video sequences of around 9 seconds duration (≈ 540 frames) each, consisting of resting and flying mosquitoes, were used for the analysis. Video sequence duration was selected by considering the light consistency and the number of flying mosquitoes and their flight patterns (covering different flight trajectories) as mosquitoes spend the majority of their time in the rest position. The frame sizes of video sequences were 640 in width and 512 in height, while the frame rates were 60 frames per second.

### Training and validation data

Training and validation were performed by using 100 images extracted from training and testing videos. Of these 100 images, 80 were used for training, and 20 were used for validation. In total, we trained for 25 epochs, and the detection threshold was kept at 70%, which means the proposals with less than 0.7 confidence were ignored. Training and validation data annotations were created with the help of VGG Image Annotator [41] in the form of.json files.

### Groundtruth data collection

The groundtruth values were calculated manually using the cursor position to check pixel values through a GitHub-based image viewer [42].

### Interpolation

Considering the simplicity and usefulness, the SRS1 Cubic Spline function (Version 2.5), which is a Microsoft Excel Add-in [43], was used to perform the cubic spline interpolation. A cubic spline interpolates a smooth line that directly passes through all points in the data set. Mainly, cubic spline interpolation tries to make the resultant curve smooth and continuous at each data point by fitting a series of cubic polynomials. This fitting process requires the matching of the first and second derivatives of the polynomials at each data point and imposing boundary conditions at the endpoints of the resultant curve.

### Metric based evaluation

Performance metrics are powerful tools used to evaluate the usability of any product. Measuring performance is a key to evaluating how well the algorithm performs its function. Considering the small sizes of mosquitoes, the performance of the proposed system is evaluated by using a custom metric that considers the three indicators: mean of distances between positions, pooled standard deviation, and average accuracy. The mean of distances between positions tells about the mean of differences in pixels between corresponding estimated central positions of mosquitoes and ground truth centroids in each frame. The formula to calculate the mean squared distance is derived from the L2-norm distance, also known as Euclidean distance. In our scenario, L2-norm distance computes the square root of the sum of the squared differences between the position of mosquitoes across the frames (Eq 1), which is then used to calculate the mean squared distance (Eq 2).


$$d_{f}=\sqrt{{{{(p}_{f}-x_{f})}^{2}+{(q}_{f}-y_{f})}^{2}}$$ (1)

$$m_{1}=\frac{1}{n}\sum_{f=1}^{n}d_{f}$$ (2)

Where *d_f_* is the distance between estimated x-axis pixels (*p_f_*), ground truth x-axis pixels (*x_f_*), and estimated y-axis pixels (*q_f_*), ground truth y-axis pixels (*y_f_*). The value of *f* shows the frame number, and *n* shows the total number of frames.

Pooled standard deviation is the weighted average of standard deviations between estimated and ground-truth trajectories data for all mosquitoes present in a video sequence, while accuracy is defined as the closest possible trajectory points captured by the mask RCNN algorithm and interpolation to the ground-truth trajectory points. The accuracy tolerance was set at 8 pixels which means if the estimated points were within 8 pixels (absolute value of both x-axis and y-axis) of ground truth centroids, they were considered as part of accuracy, as expressed in expression (3). This was based on the fact that the mosquito is not a single pixel organism, and it could be sitting in any position, so it was not possible to estimate the exact centroids of mosquitoes. The value of 8 pixels was selected by taking the average length of 10 randomly selected mosquitoes which was 1.25% of the x-axis and 1.56% of the y-axis. The logical expression considered for the accuracy is given below.


$$if\;\left\{ \begin{array}{c} {|(p}_{f}-x_{f})|<8\cap |{(q}_{f}-y_{f})|<8\;Accurate\\ else\;Inaccurate \end{array}\right.$$ (3)

Custom metric performs the quantitative assessment, and it takes the position of estimated values and compares them with the ground truth values to calculate indicators values.

### Tracker error based verification

Tracker error is used to perform an in-depth performance evaluation of any algorithm. In the experiments, tracking error is the point to point difference in pixels between mosquitoes’ estimated centre positions and ground truth centroids in each frame. The tracker error was calculated by using Eq 1. In tracker error-based verification, only flying mosquitoes were considered, as the accuracy for the sitting mosquitoes was 100% in all case scenarios.

### Programming and computational system

All programming and computational analyses were performed on a laptop computer, run under a 64-bit Windows 10 Pro environment using Intel i7-10510U (1.80 GHz and 2.30 GHz) processor and 16 Gb DDR4 RAM. The method was implemented in Python 3.7, OpenCV 3.3.1, Jupyter Notebook 6.4.2, and Tensorflow 1.14.0. The details of other libraries and their versions are available in the Requirements File in S1 File.

### Trajectory estimation using machine learning algorithm

Mosquitoes’ detection and trajectory estimation was performed with the help of a custom-developed technique, which uses the Mask RCNN algorithm and spline interpolation. Mask RCNN is a deep neural network that helps to extract different objects from an input image or video. Mask RCNN is the extension of Faster RCNN and uses open-source libraries of Keras and Tensorflow. The Mask RCNN model used in the experiment is based on Feature Pyramid Network (FPN) and a ResNet101 backbone [44]. A Feature Pyramid Network is a feature extractor that facilitates the creation of multiple feature map layers with quality information. ResNet101 is a convolutional neural network having 101 layers. These layers help to improve accuracy and performance as each layer can learn complex features such as detecting edges and identifying textures. The trajectory estimation method involving the Mask RCNN framework is shown in Fig 1B.

Matterport Mask RCNN’s existing model (based on the MSCOCO dataset) [45] could not track the mosquitoes; therefore, custom training was performed for mosquito detection (their location), mosquito localisation (their extent), and instance segmentation (boundaries identification at detailed pixel level) of mosquito containing images. Jupyter Notebook was used to run the code, perform validation and load the videos to the algorithm. The mask RCNN available code could process the images and spot the locations of the objects in each image; however, in our case of feeding videos using OpenCV, we were looking to automatically extract the pixel locations of corresponding mosquitoes in each frame to draw the trajectories with the less work. Therefore, the existing code was modified to automatically identify the locations of each mosquito in each frame. The method monitors the connectivity of mosquito pixel locations in consecutive frames as well as the trajectory’s direction to ensure that it is the continuation of the previous trajectory. If a mosquito was detected in a frame, then in the next frame algorithm looked for the same mosquito at the nearest distance by comparing the x-axis and y-axis pixels’ locations of all mosquitoes with the locations of mosquitoes in the previous frame. The algorithm stored the data of each mosquito in the form of text files. For instance, if the detected pixels’ location of mosquito 2 in frame number 400 is 123 width, and 249 height (x-axis and y-axis values, respectively), then the algorithm will compare it with the locations of all mosquitoes in the previous frame and based on the difference in distances will store it in the text file of mosquito 2. The data that the algorithm misses due to background clutters can be filled through interpolation. Interpolation generates the missing data in a smooth form by using known data points.

**Fig 2:**
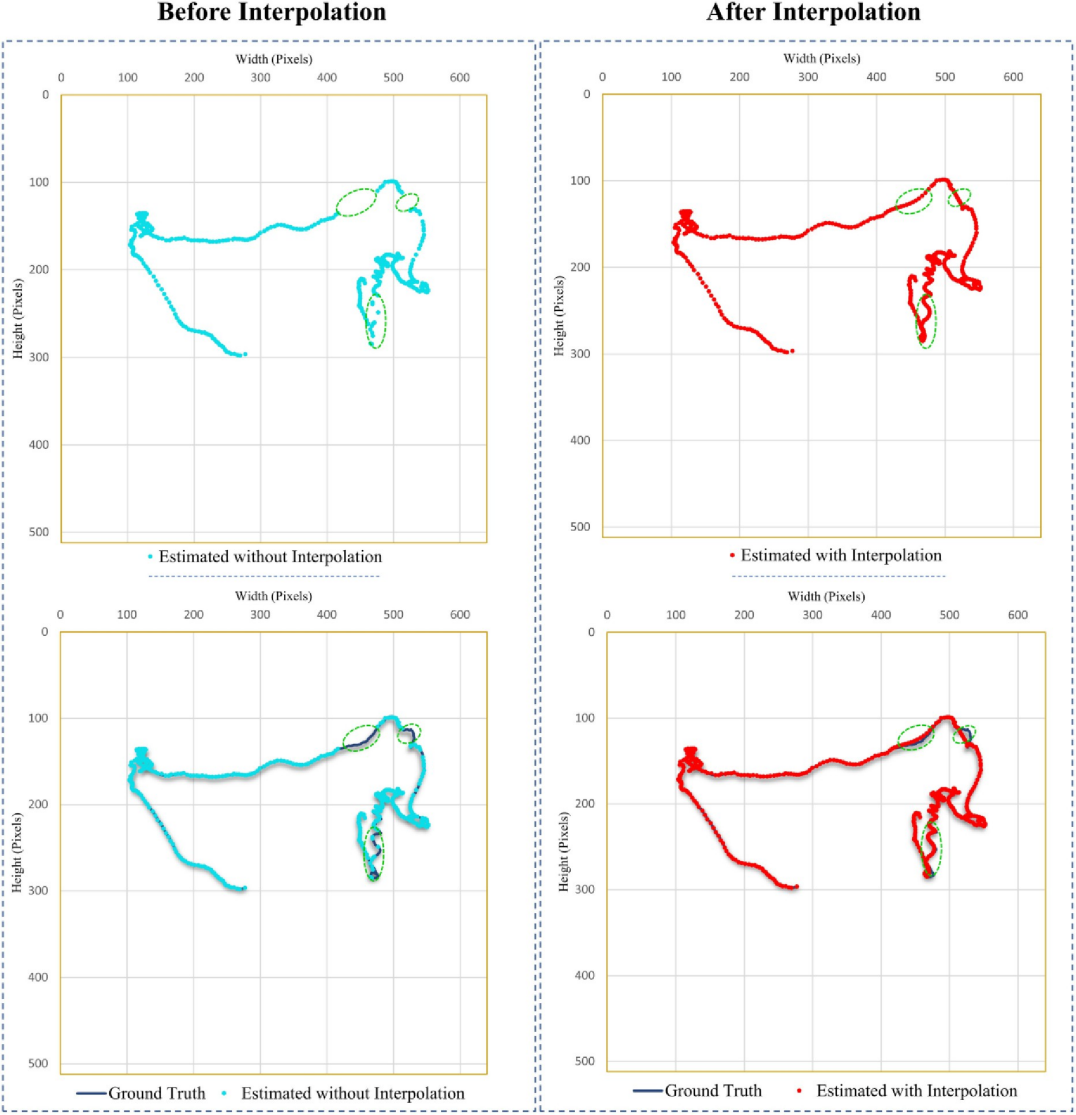
Impact of interpolation on trajectory estimation. In the top 2 charts, the impact of interpolation with and without interpolation is shown individually, while in the bottom graphs, they are shown along with ground truth trajectories. It can be seen from the green dotted circled areas that spline interpolation helped to fill the missing points and achieve continuous tracking with higher accuracy.

In our case, the data obtained from the algorithm was missing the location of mosquitoes in the frames where mosquitoes faced the background clutters and light reflection. Mosquitoes follow arbitrary flight patterns; therefore, a nonlinear curve fitting method, cubic spline interpolation, which worked perfectly in our scenario, was used to fill the data in the frames where mosquitoes’ locations were not detected. Cubic spline interpolation is a mathematical technique generally used to generate new data points within the boundaries of known data. In cubic spline interpolation, unique cubic polynomials are fitted between each data point, with the condition that the curve obtained after interpolation be continuous and look smooth. Fig 2 shows the impact of spline interpolation on a mosquito flight trajectory. The accuracy without spline interpolation was 84.57%, while the accuracy with spline interpolation was 98.69%.

## Results

This section presents the experimental results obtained after feeding the videos to the algorithm and performing the spline interpolation to the algorithm’s output. The results are obtained from three different video sequences and presented in the form of case scenarios depending on the number of flying mosquitoes and the total number of mosquitoes present in the cage.

### Case scenario 1: Two flying mosquitoes and five total mosquitoes in the cage

**Fig 3:**
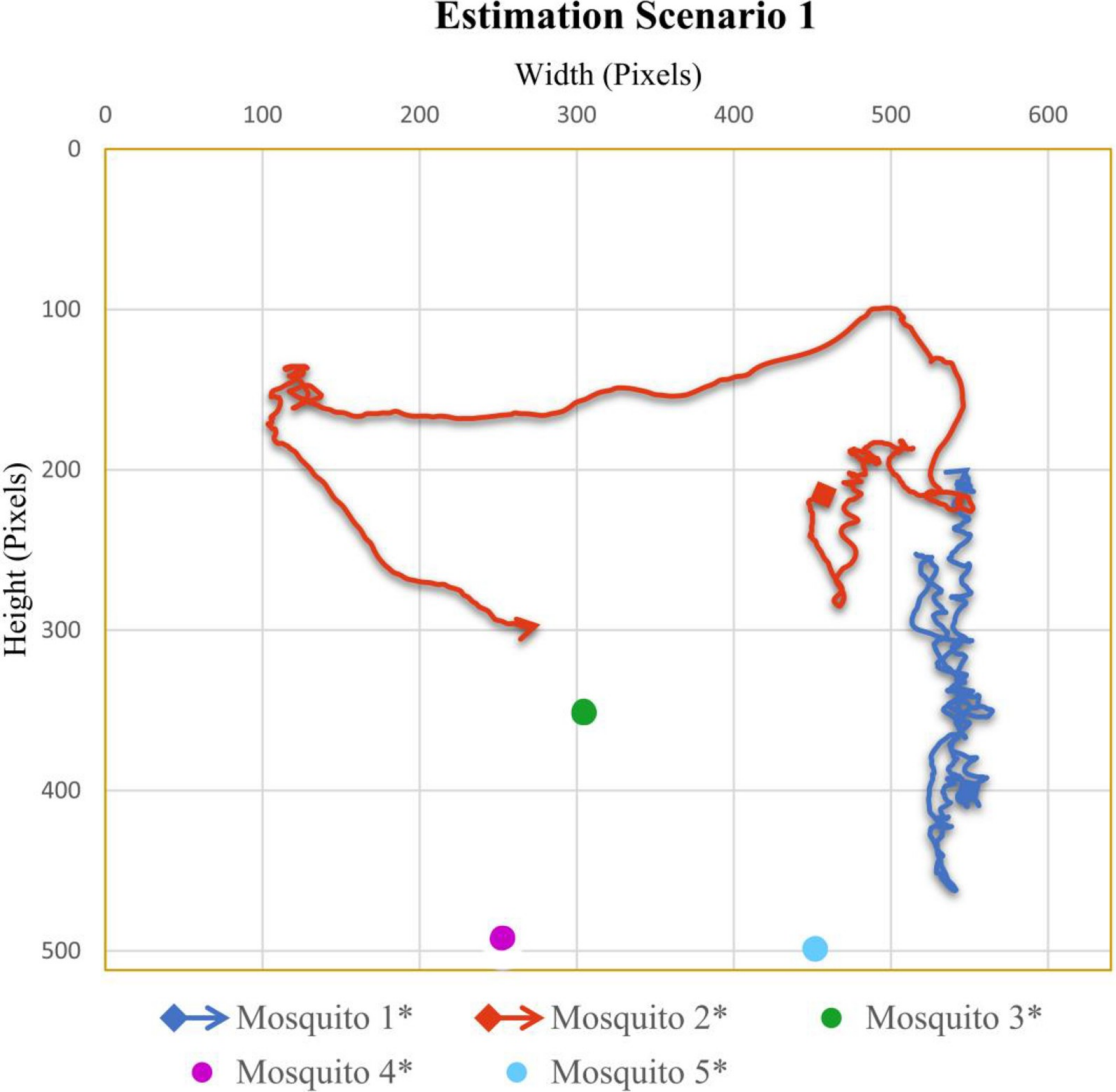
Scenario 1 results obtained from Mask RCNN and interpolation. Flying mosquitoes’ flight starting points are shown with dots, while the flight endpoints are shown with arrows. The mosquitoes in the ‘rest’ position are presented with filled marker dots. To distinguish the ground truth trajectories from estimated trajectories which will be discussed in the next section, the names of the mosquitoes for estimated trajectories are indicated with asterisk symbols. Different colours are also used to distinguish the mosquitoes from each other.

In the video sequence of scenario one, the total number of mosquitoes was 5, out of which 2 were flying. In Fig 3, the flight trajectory and rest position of different mosquitoes present in video sequence one are given. Mosquito 2 kept on flying in a certain area, while mosquito 1 covered most of the cage area.

### Case scenario 2: Three flying mosquitoes and six total mosquitoes in the cage

**Fig 4:**
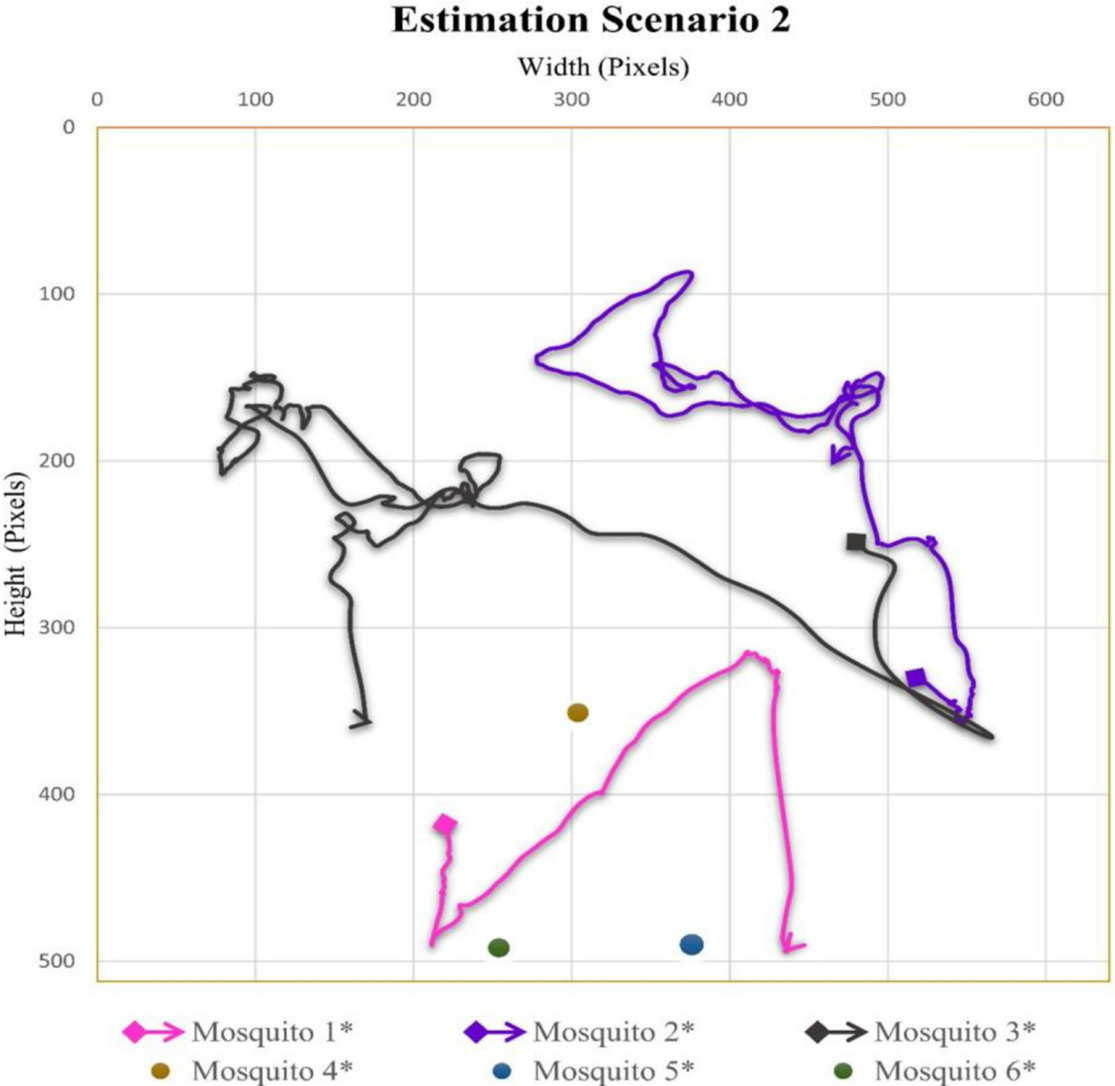
Scenario 2 results obtained from Mask RCNN and interpolation.

In the video sequence of scenario two, the number of flying mosquitoes was three, while the total number of mosquitoes was 6. In Fig 4, the flight trajectory and rest position of different mosquitoes present in video sequence two are given. In video 2, the mosquito 3 flight covered the maximum area of the cage compared to mosquitoes 1 and 2.

### Case scenario 3: One flying mosquito and twenty-seven total mosquitoes in the cage

**Fig 5:**
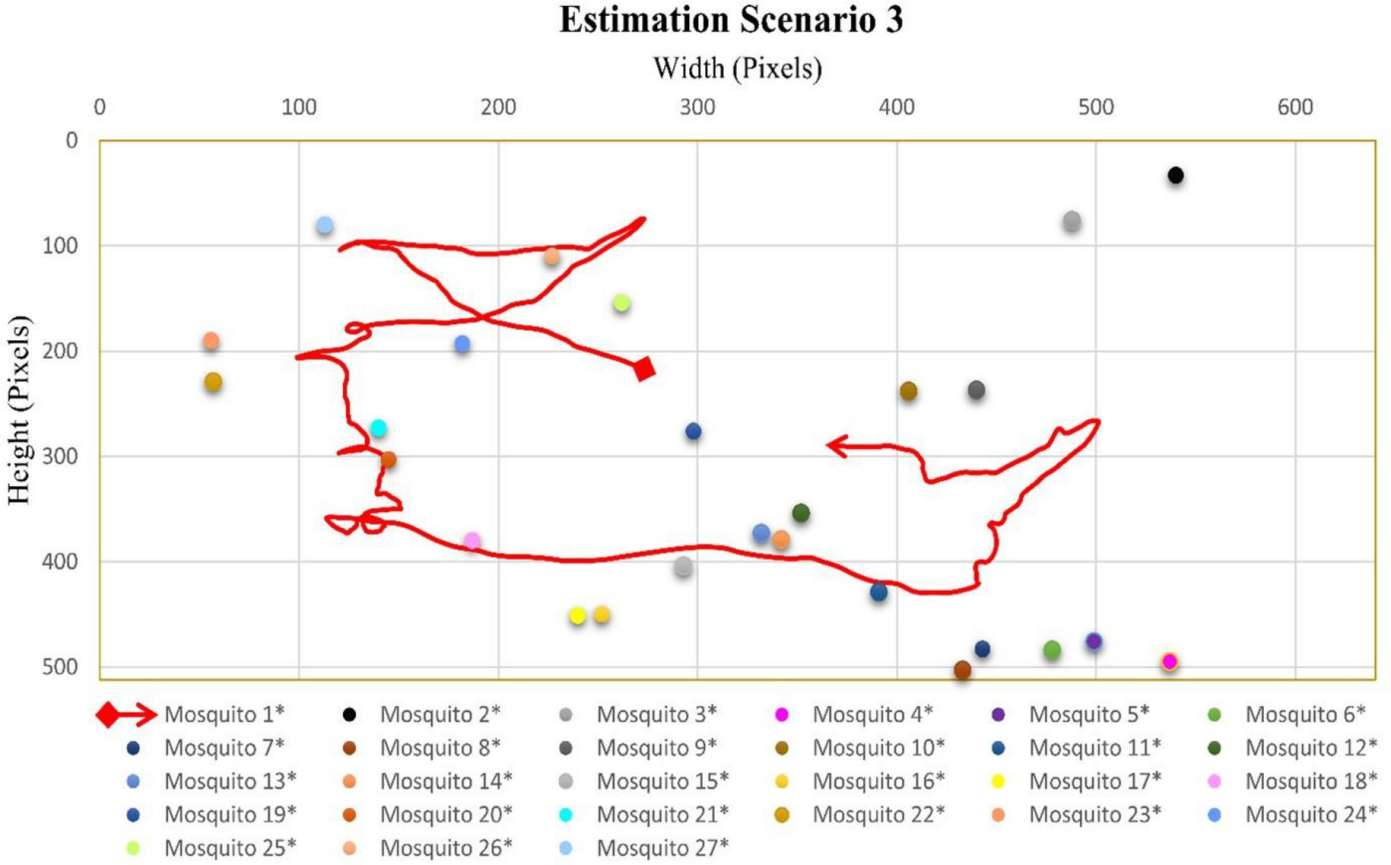
Scenario 1 results obtained from Mask RCNN and interpolation.

In the video sequence of scenario three, the number of flying mosquitoes was 1, while the total number of mosquitoes was 27. In Fig 5, the flight trajectory and rest position of different mosquitoes present in video sequence three is given. Mosquito 1 flight started from almost the middle of the cage and flew between most of the sitting mosquitoes.

## Performance evaluation

The proposed method was validated by using a custom metric and tracker error analysis. The following subsections present the metric-based verification and tracker error-based analysis.

### Metric based evaluation

#### Case scenario 1: Two flying mosquitoes and five total mosquitoes in the cage

**Fig 6:**
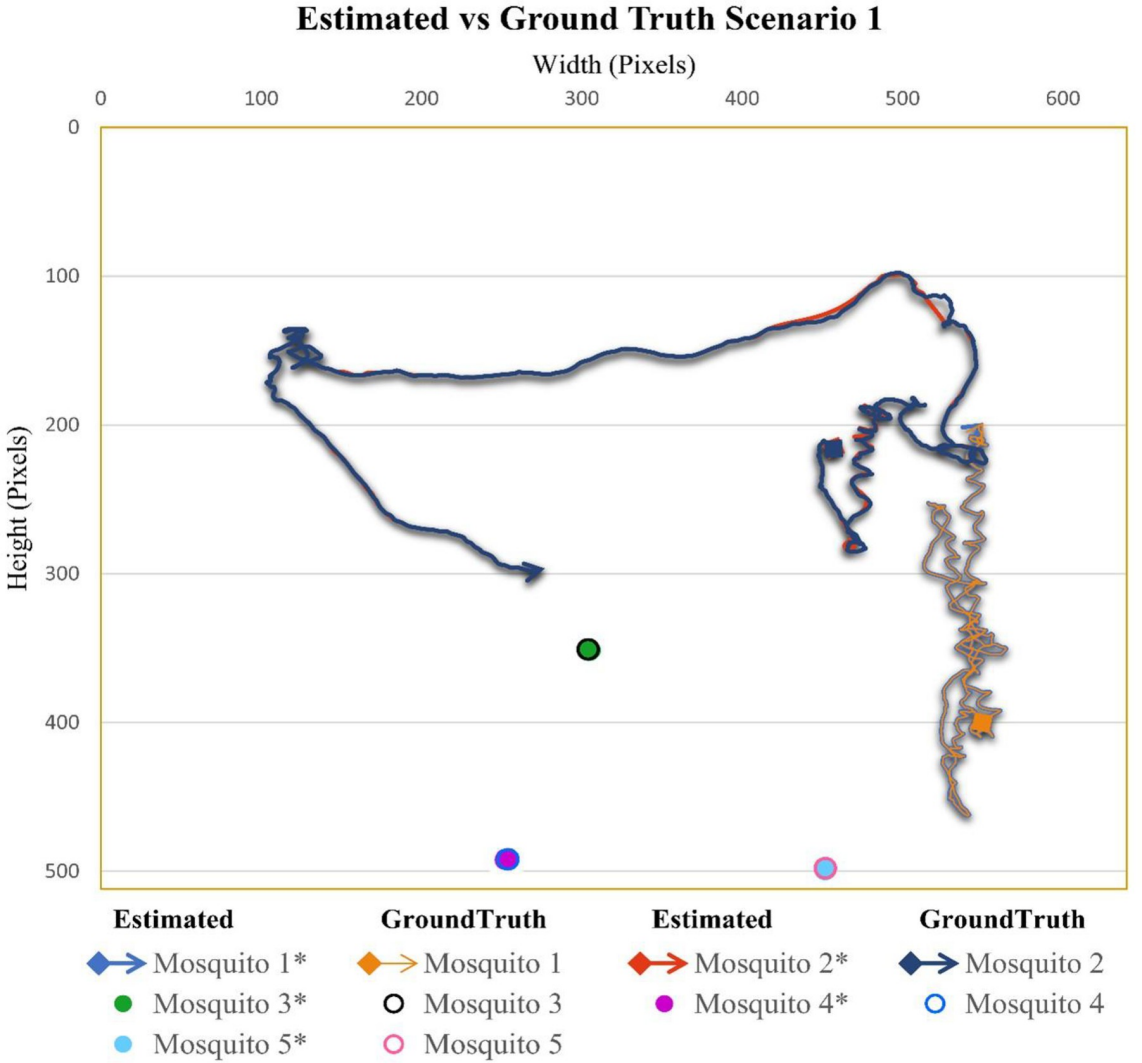
Comparison of estimated and ground-truth values for scenario 1.

In video sequence 1, the mean distance between positions (distance between central positions of mosquitoes and ground truth centroids) was 0.66 pixels, while the pooled standard deviation, which is the combined standard deviation of all mosquitoes present in video 1, was 0.79. There were two flying mosquitoes in video sequence 1. Detection accuracies for mosquitoes 1 and 2 were 100% and 98.69%, respectively, while the overall accuracy for flying mosquitoes was 99.35% (Table 1). The accuracy for mosquitoes in the rest position was 100%. The combined accuracy for flying and sitting mosquitoes was 99.73%. Fig 6 shows the comparison between estimated trajectories (for flying mosquitoes) and positions (for sitting mosquitoes) and ground truth trajectories and positions. We can observe that the proposed method could successfully detect and track the flying and sitting mosquitoes as there are minor differences between the trajectories. The areas where there are small differences between the estimated and ground-truth trajectories consist of the data points where the mosquitoes were facing background clutters (cage boundary, feeding bottle, dark patches on background fabric net) and light reflection.

**Table 1:** Performance evaluation metric scenario 1.

Scenario	Number of Frames	Total Mosquitoes in Cage	Flying Mosquitoes in Cage	Mean Distance Between Positions (Pixels)	Pooled Standard Deviation	Sitting Mosquitoes Accuracy	Mosquito 1 Accuracy	Mosquito 2 Accuracy	Average Accuracy
1	538	5	2	0.66	0.79	100%	100%	98.69%	96.62%

#### Case scenario 2: Three flying mosquitoes and six total mosquitoes in the cage

**Fig 7:**
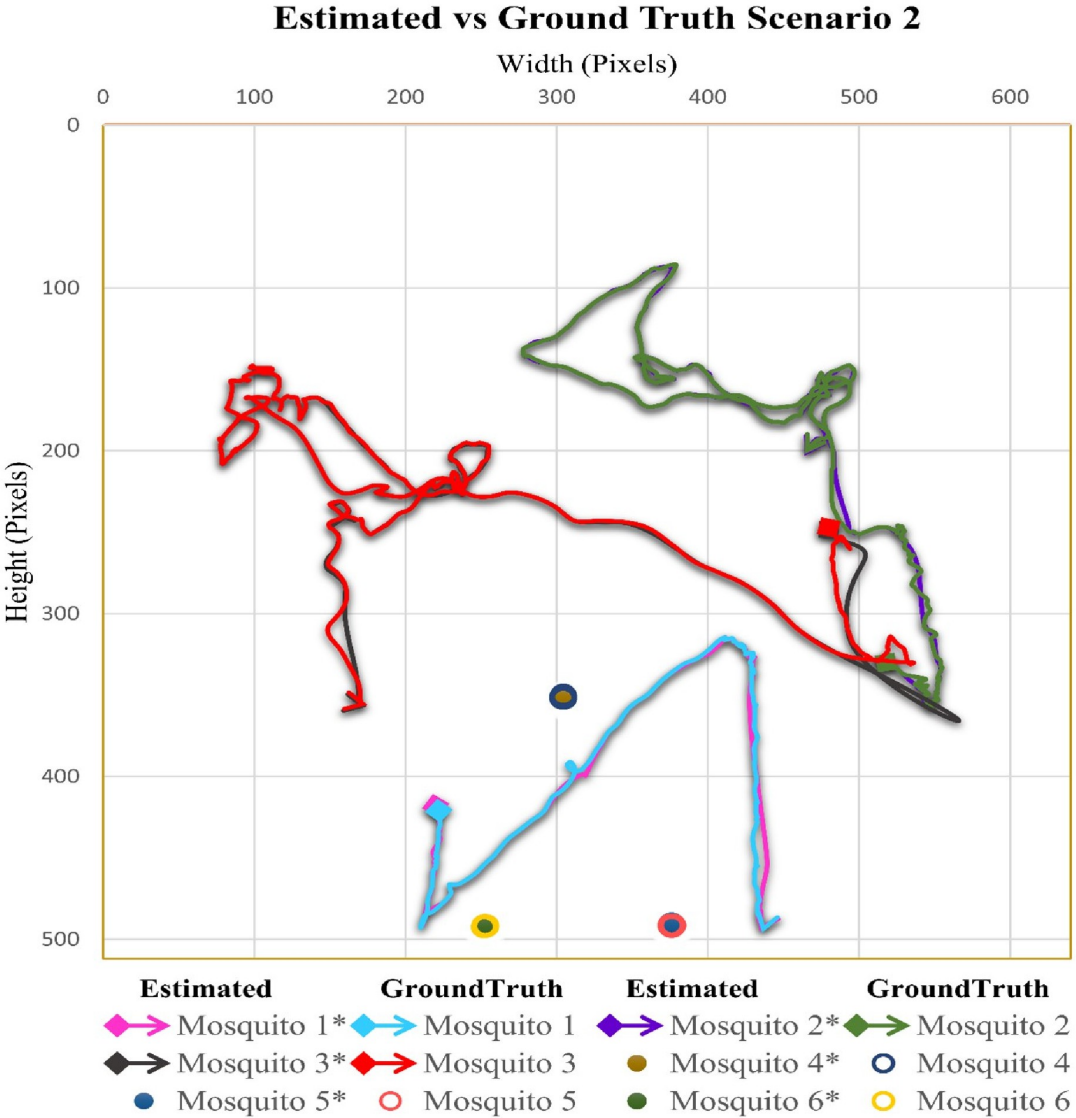
Comparison of estimated and ground-truth values for scenario 2.

In video sequence 2, the mean distance between positions was 1.83 pixels, while the pooled standard deviation was 4.63. There were three flying mosquitoes in video sequence 2. Detection accuracies for mosquitoes 1, 2, and 3 were 91.34%, 99.31%, and 89.62%, respectively, while the overall accuracy for flying mosquitoes was 93.42% (Table 2). The accuracy for mosquitoes in the rest position was 100%. The combined accuracy for flying and sitting mosquitoes was 96.731%. Fig 7 shows the comparison between estimated flight trajectories and sitting positions and ground truth flight trajectories and sitting positions.

**Table 2:** Performance evaluation metric scenario 2.

Scenario	Number of Frames	Total Mosquitoes in Cage	Flying Mosquitoes in Cage	Mean Distance Between Positions (Pixels)	Pooled Standard Deviation	Sitting Mosquitoes Accuracy	Mosquito 1 Accuracy	Mosquito 2 Accuracy	Mosquito 3 Accuracy	Average Accuracy
2	578	6	3	1.826	4.63	100%	91.34%	99.31%	89.62%	96.71%

#### Case scenario 3: One flying mosquito and twenty-seven¬ total mosquitoes in the cage

**Fig 8:**
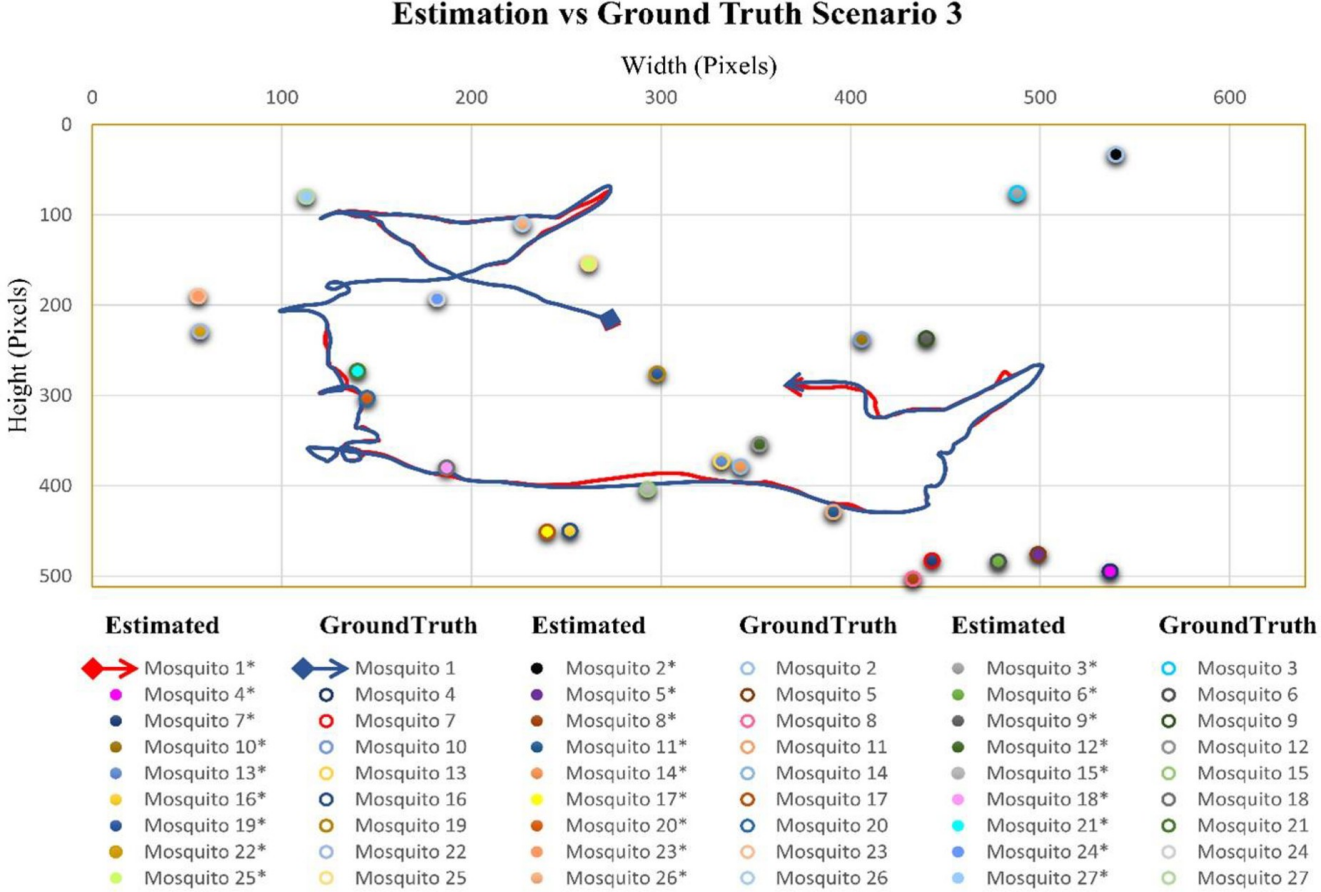
Comparison of estimated and ground-truth values for scenario 3.

In video sequence 3, the mean distance between positions was 1.47 pixels, while the pooled standard deviation was 0.54. There was one flying mosquito in video sequence 3. The detection accuracy for flying mosquitoes was 95.58%. The accuracy for mosquitoes in the rest position was 100%. The combined accuracy for flying and sitting mosquitoes was 99.83% (Table 3). Fig 8 shows the comparison between estimated flight trajectories and sitting positions and ground truth flight trajectories and sitting positions.

**Table 3:** Performance evaluation metric scenario 3.

Scenario	Number of Frames	Total Mosquitoes in Cage	Flying Mosquitoes in Cage	Mean Distance Between Positions (Pixels)	Pooled Standard Deviation	Sitting Mosquitoes Accuracy	Mosquito 1 Accuracy	Average Accuracy
3	541	27	1	1.47	0.54	100%	95.58%	99.83%

### Tracker error based verification

#### Case scenario 1: Two flying mosquitoes and five total mosquitoes in the cage

**Fig 9:**
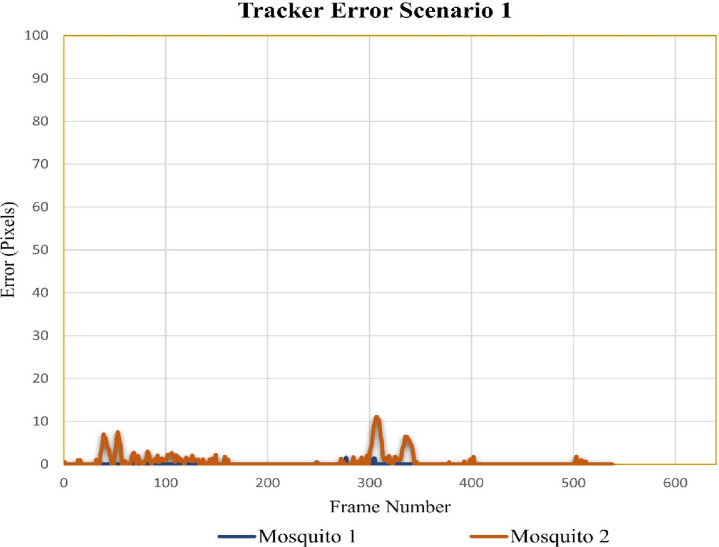
Error tracking for scenario 1.

In video sequence one, the total number of frames was 538. Fig 9 shows the tracker error for video sequence one where mosquitoes 1 and 2 were flying. For mosquito number 1, the tracking error was very low in all frames, which shows that it very accurately tracked the trajectory of mosquito 1. For mosquito 2, tracking errors are negligible in most areas; however, some minor differences can be observed around frames 304 to 310. The minor differences were due to the flight of mosquito 2 in the background dark patches.

#### Case scenario 2: Three flying mosquitoes and six total mosquitoes in the cage

**Fig 10:**
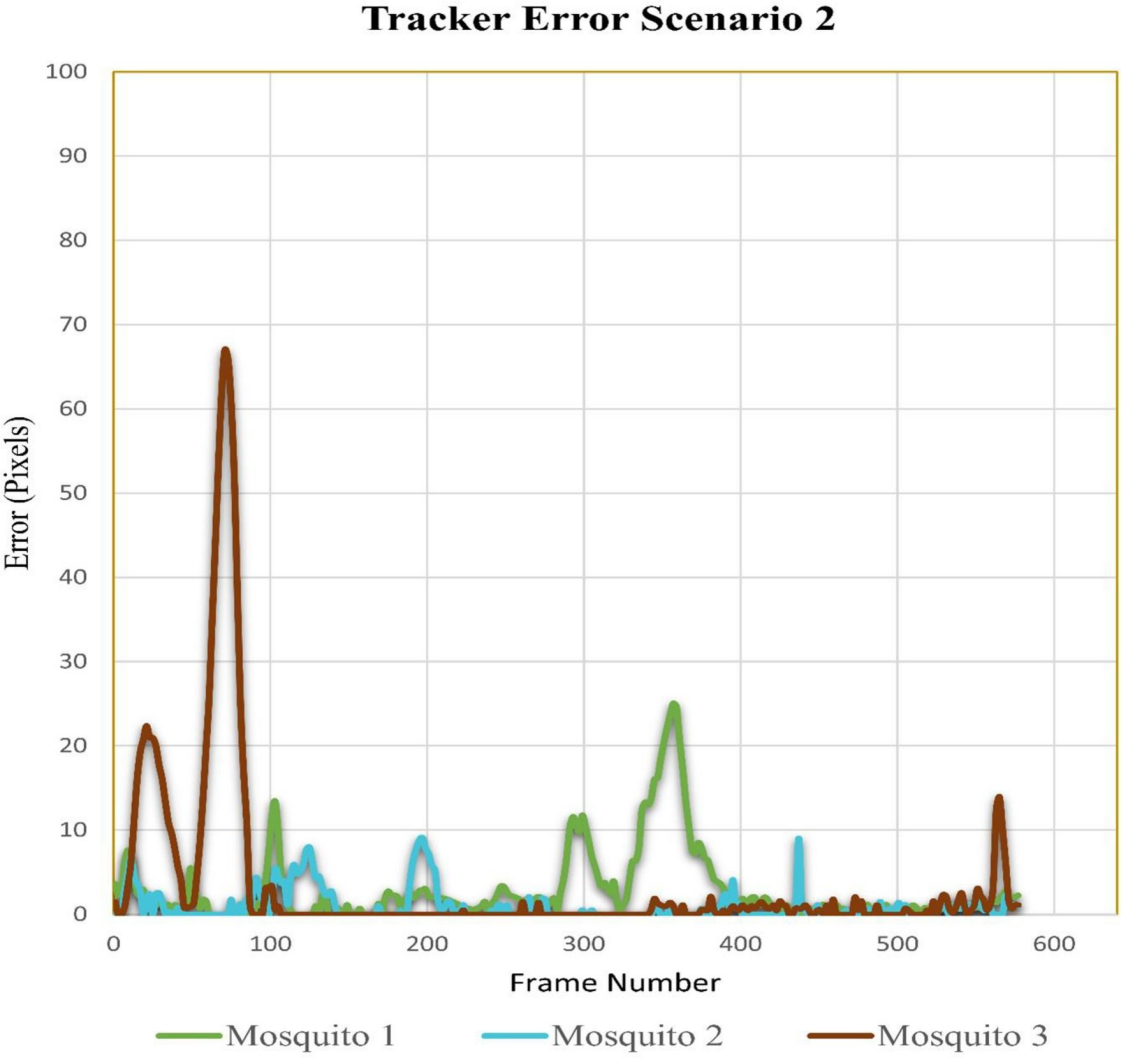
Error tracking for scenario 2.

In video sequence two, the total number of frames was 578. For mosquito number 2, the tracking errors were very low, while for mosquitoes 1 and 3, they were high in a few frames. Fig 10 shows the tracker error for flying mosquitoes 1, 2, and 3. For mosquito 1, some differences between estimated and ground truth can be observed around frames 293 to 297 and 337 to 365, while for mosquito 3, differences can be observed around frames 13 to 35 and 57 to 83. Higher tracker error for mosquito 3 around frames 57 to 83 was due to its continuous flight in background dark net folds.

#### Case scenario 3: One flying mosquito and twenty-seven total mosquitoes in the cage

**Fig 11:**
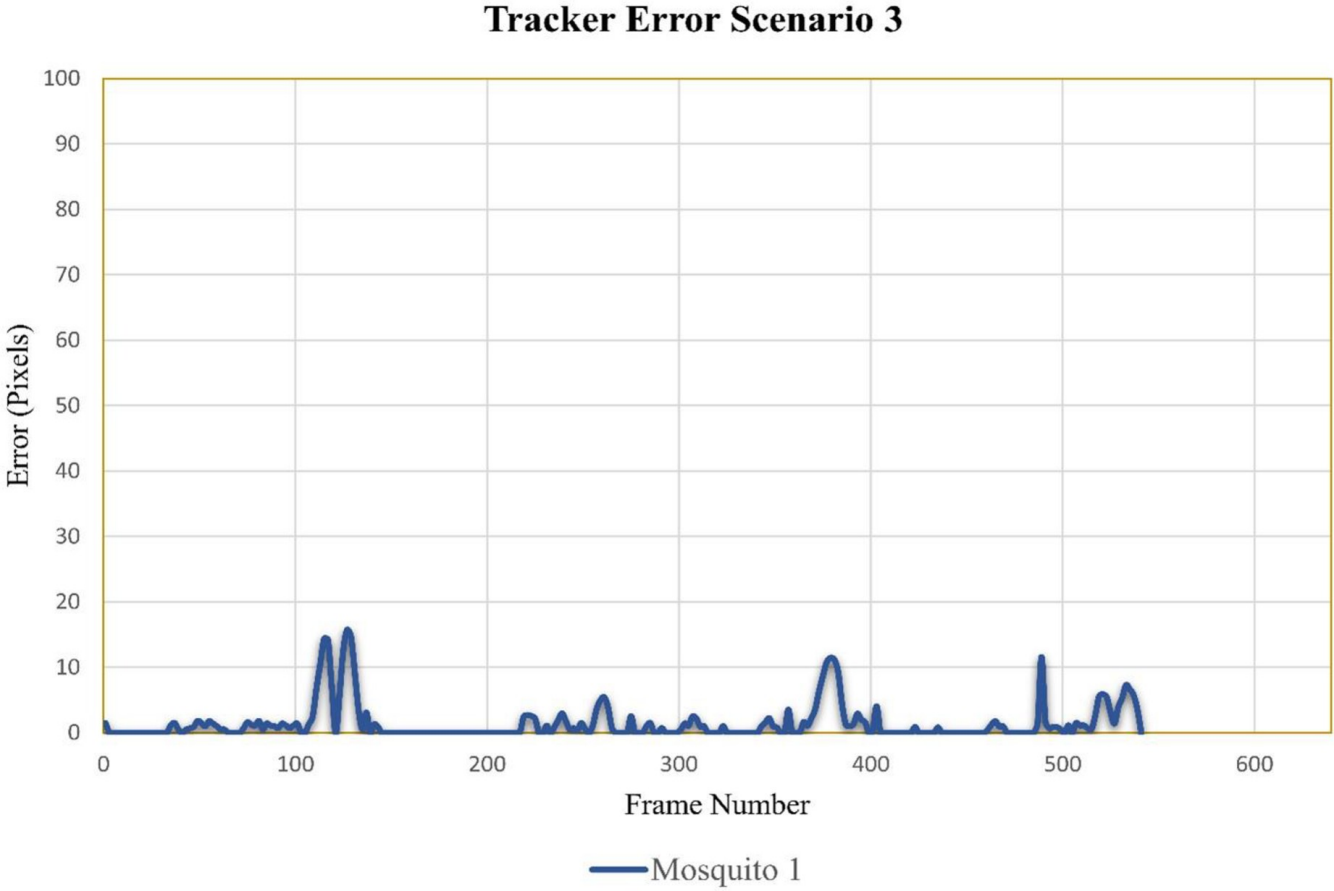
Error tracking for scenario 3.

In video sequence three, the total number of frames was 541. For mosquito 1, some differences can be observed around frames 113 to 117 and 125 to 129 (Fig 11). These differences were due to its flight around dark lines of cage boundary and feeding sugar bottle. The overall results showed that this method could very precisely track the trajectory of mosquitoes.

## Discussions

Though the method has shown excellent results in tracking mosquitoes’ trajectory, however, there are also some limitations of this method. If there are significant gaps between the data points for different reasons, including background distortions and light reflection, cubic spline interpolation makes interpolated values inaccurate by several orders of magnitude. Eventually, making the curve too complex and not helpful in making predictions. In such scenarios, other interpolation methods might be considered, such as linear interpolation or polynomial interpolation of a lower order.

In the case of occlusions (mosquitoes crossing each other), if mosquitoes deviate slightly after the occlusion, they can be detected successfully by looking at the connectivity of mosquito pixel locations in consecutive frames and the direction of the trajectory through the model and interpolation. If the diversion is at a higher angle, then manual observation of the crossing mosquitoes will be required for the frames where they cross each other. However, to make the manual corrections process easy, the feature of locating frame numbers was also added in the code; output text files contain the frame numbers along with axis data; therefore, it is easy to locate the errors and make the manual corrections. The algorithm can also generate more than one file for each mosquito depending on the background distortions and light reflection; however, combining the data of different files will be effortless by looking at the starting and last values of frame numbers and axis values of each file.

This work can benefit mosquito flight behaviour monitoring and quantification related studies as the trained model can perform well with similar kinds of setups or even if there are slight changes in the setup. When the model was applied to another video (see Video 4 in S1 File) having a slightly different setup with smaller cage dimensions 25x25x25, no sugar water bottle, and white non-fabric background, the method still detected all the mosquitoes. However, bespoke training for different setups can improve the results further.

## Conclusions

Detection and flight tracking is important in studying the behavioural traits of mosquitoes. Small sizes, complicated poses, and seemingly arbitrary moving patterns create many different challenges for successfully tracking mosquitoes. This paper presents a trajectory extraction method that utilises the Mask RCNN detection algorithm and cubic spline interpolation for standard laboratory environment videos. Three case scenarios covering different flight trajectories were used for the verification. Metric and tracker error-based verification showed that the presented method is an excellent option for mosquito monitoring and could efficiently track the mosquitoes present in a video, even if they have a similar texture compared to the background. The results were comparable to manually calculated ground truth values, and the average accuracy of three case scenarios was 96.62%, 96.71%, and 99.83%, respectively. The performance can be improved further by increasing the number of training images.

This algorithm is the one step towards developing an automatic mosquito behaviour monitoring system. The development of such methods is vital for determining the fitness of infected or modified mosquitoes and will be useful in vector-borne disease modelling and the development of novel mosquito traps.

## Supporting information

S1 File (RAR)
